# Efficacy and safety of a reduced calcineurin inhibitor dose combined with mycophenolate mofetil in liver transplant patients with chronic renal dysfunction

**DOI:** 10.18632/oncotarget.15490

**Published:** 2017-02-18

**Authors:** Pusen Wang, Weitao Que, Hao Li, Lvnan Yan, Zhiren Fu, Qifa Ye, Guihua Chen, Kefeng Dou, Shichun Lu, Zhanyu Yang, Zhijun Zhu, Zhihai Peng, Lin Zhong

**Affiliations:** ^1^ Department of General Surgery, Shanghai General Hospital, Shanghai Jiao Tong University School of Medicine, Shanghai, China; ^2^ Department of Liver Surgery and Liver Transplantation Center, West China Hospital, Sichuan University, Chengdu, China; ^3^ Organ Transplantation Institute of Changzheng Hospital, Second Military Medical University, Shanghai, China; ^4^ Engineering and Technology Research Center for Transplantation Medicine of the National Ministry of Health, The Third Xiangya Hospital, Central South University, Changsha, China; ^5^ Department of Liver Transplantation, Third Affiliated Hospital, Sun Yat-sen University, Guangzhou, China; ^6^ Department of Hepatobiliary Surgery, Xijing Hospital, The Fourth Military Medical University, Xian, China; ^7^ Department of Liver Transplantation, Beijing Youan Hospital, Capital Medical University, Beijing, China; ^8^ Department of Hepatobiliary Surgery, Southwest Hospital, Third Military Medical University, Chongqing, China; ^9^ Department of Transplantation, Tianjin First Central Hospital, Tianjin Medical University, Tianjin, China

**Keywords:** calcineurin inhibitors, mycophenolate mofetil, liver transplantation, efficacy, safety

## Abstract

Calcineurin inhibitors (CNIs) are frequently given at a reduced dose in combination with mycophenolate mofetil (MMF) to avoid nephrotoxicity, but the optimal reduction in CNI dose has not been established. In this prospective, open-label, multicenter study, liver transplant recipients with chronic renal dysfunction who were administered a CNI-based immunosuppressive regimen were included in the intent-to-treat (ITT) population. The primary endpoint was declination in renal function, which was defined as a ≥ 20% decrease in the glomerular filtration rate during the year following regimen adjustment. In the ITT population, renal function declined after regimen adjustment in three patients (7%) in the MMF plus 50% CNI reduction group. Additionally, three of 40 patients (7.5%) in the MMF plus 75% CNI reduction group experienced at least one clinically suspected or biopsy-proven acute rejection. There were no differences between the two groups. The corrected mean improvement in creatinine clearance at week 52 was 6.551 mL/min in the MMF plus 50% CNI reduction group and 6.442 mL/min in the MMF plus at least 75% CNI reduction group. Thus, a regimen of MMF combined with a 50% or at least 70% reduction in CNI dose could improve renal function and was both tolerable and safe.

## INTRODUCTION

Liver transplantation (LT) is a highly successful treatment for end-stage liver disease and select hepatocellular carcinoma patients. Approximately 70% or more of LT recipients survive for at least 5 years post-transplant [1, 2]. However, renal dysfunction is a major late complication and is a cause of poor long term prognosis after LT [3–7]. Chronic renal failure in non-renal transplant recipients increases the risk of death by more than four-fold [4]. Hypertension, diabetes, treatment with immunosuppressants, hepatitis C virus infection, and calcineurin inhibitor (CNI) toxicity are frequent comorbidities in LT recipients that can lead to renal dysfunction.

CNI toxicity is the primary cause of chronic renal dysfunction [3, 4, 8, 9]. CNIs are frequently used as first-line immunosuppressive agents in LT patients [10]. CNIs have improved both graft and patient survival rates [11]. CNI dose-minimizing regimens are currently under investigation to avoid acute and chronic nephrotoxicity [9, 12–14]. Mycophenolate mofetil (MMF) is one of the most commonly used non-nephrotoxic immunosuppressants [15]. Previous studies have demonstrated that it can reduce CNI-related toxicities [12, 16–22]. In a prospective, multicenter, randomized study performed by Pageaux et al. [12], MMF in combination with at least a 50% reduction in CNI dose significantly improved renal function in LT recipients at 1 year without causing rejection. No improvement in renal function was observed in LT recipients who received a less than 25% reduction in CNI dose without the addition of MMF. In another prospective, randomized pilot trial, Gerhardt et al. [23] demonstrated that MMF in combination with a 75% dose reduction in CNI could improve the glomerular filtration rate (GFR) in LT recipients with moderately elevated serum creatinine (SCr) levels.

MMF monotherapy for CNI-related toxicity is controversial. This is because some studies have reported that substitution of CNI with MMF was associated with an increased risk of rejection or toxicity [15, 24], while others have shown that it was safe and effective [25, 26]. Although MMF in combination with either a 50% or 75% CNI dose reduction is safe and effective for chronic renal dysfunction following LT, the most effective regimen has not been established. We evaluated whether the conversion of LT recipients with chronic renal dysfunction to treatment with MMF in combination with CNI at a reduced dose (e.g. 50% or 75%) could improve renal function in LT recipients without increasing the risk of rejection or adverse events (AEs). We hypothesized that these regimens could also reduce the incidence of hypertension, neurotoxicity, glucose intolerance, hyperlipidemia, and gastrointestinal side effects.

## RESULTS

### Patient disposition

**Figure 1:**
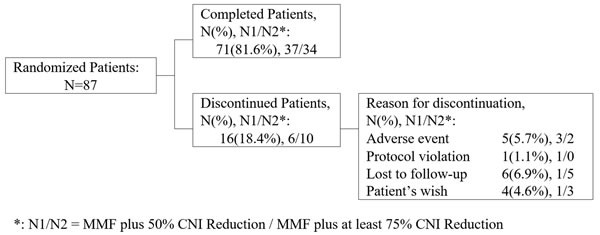
Overview of the analysis populations *: N1/N2 = MMF plus 50% CNI reduction / MMF plus at least 75% CNI reduction.

A total of 87 patients from nine centers in China were randomized and enrolled in this study. There were 71 patients (81.6%) who completed the study and 16 who did not (6 patients in the MMF plus 50% CNI reduction group and 10 in the MMF plus at least 75% CNI reduction group). No patients died during the study period. Five patients (5.7%) discontinued due to AEs and one due to a protocol violation. An additional 10 patients withdrew from the study for personal reasons (lost to follow-up or at their own wish) (Figure 1). The intent-to-treat (ITT) population consisted of 83 patients (43 in the MMF plus 50% CNI reduction group, and 40 in the MMF plus at least 75% CNI reduction group). Four patients were excluded because they were not taking any of the study drugs. Sixteen patients were removed from the per-protocol (PP) population due to discontinuation, and 71 were included in the PP population (37 in the MMF plus 50% CNI reduction group, and 34 in the MMF plus at least 75% CNI reduction group).

### Demographic and other baseline characteristics

The demographic and selected baseline characteristics of the PP population are shown in Table 2. Ninety percent of the patients were male and the mean age was 53 years in both treatment groups. The mean calculated creatinine clearance at baseline in the MMF plus 50% CNI reduction group was significantly lower than in the MMF plus at least 75% CNI reduction group (53.412 mL/min vs. 60.405 mL/min, respectively, *P* = 0.025). The mean serum creatinine at baseline in the MMF plus 50% CNI reduction group was significantly higher than in the MMF plus at least 75% CNI reduction group (133.32 μmol/L vs. 122.64 μmol/L, respectively, *P* = 0.044). The distribution of the CNI trough concentration was comparable between the two treatment groups but was not statistically significant.

**Table 1:** Secondary Endpoints

Efficacy	Safety
Rates of graft loss and patient death Biopsy-proven acute rejection after switch to MMF Change from baseline for calculated creatinine clearance after switch to MMF Change from baseline for serum creatinine after switch to MMF	All adverse evaents, including clinically significant abnormalities of clinical and laboratory parameters New onset of hypertension and cardiovascular diseases New onset of PTDM, hyperlipidemia and malignancies Incidence of opportunistic infections

**Table 2:** Demographic and Other Selected Baseline Characteristics (PP Population)

Item	MMF plus 50% CNI Reduction (*N* = 37)	MMF plus at least 75% CNI Reduction (*N* = 34)	*P* value between groups
Gender, Male (%)	89.2%	88.2%	*P* = 1.000
Age, Mean(SD) (years)	52.8(8.7)	52.4(11.1)	*P* = 0.874
Height, Mean(SD) (cm)	168.43(5.70)	170.25(7.79)	*P* = 0.263
Weight, Mean(SD) (kg)	65.34(8.99)	69.75(11.72)	*P* = 0.078
Serum creatinine (μmol/L)			*P* = 0.044
Mean(SD)	133.32(28.31)	122.64(13.12)	
Median(Min∼Max)	122.00(105.4∼232.0)	121.25(102.0∼161.2)	
Creatinine clearance (ml/min)			*P* = 0.025
Mean(SD)	53.412(13.241)	60.405(12.432)	
Median(Min∼Max)	53.138(34.94∼82.83)	60.175(31.87∼89.97)	
Trough concentration of tacrolimus (ng/ml)			*P* = 0.395
N	29	27	
Mean(SD)	6.6952(2.7266)	5.8574(4.3355)	
Median(Min∼Max)	6.4000(3.400∼15.900)	5.1000(1.600∼21.700)	
Trough concentration of cyclosporine (ng/ml)			*P* = 0.466
N	7	5	
Mean(SD)	106.563(51.337)	224.100(324.593)	
Median(Min∼Max)	118.400(30.50∼168.94)	88.900(19.30∼800.00)	

### Treatment regimens

**Figure 2:**
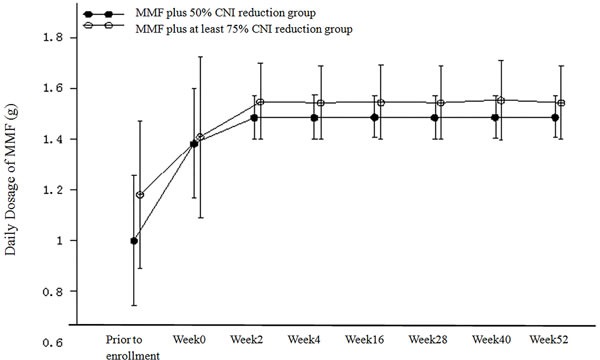
Daily Dosage of MMF (g) (PP Population)

A schematic of the transition to a daily MMF regimen in the PP population is shown in Figure 2. The mean daily dose of MMF in the MMF plus 50% CNI reduction group was slightly lower than that in the MMF plus at least 75% CNI reduction group (with statistical significance at all time points [including prior to enrollment] with the exception of week 0). The mean daily dose of MMF was 1.38 g in the MMF plus 50% CNI reduction group and 1.41 g in the MMF plus at least 75% CNI reduction group at week 0. The mean daily dose of MMF was stable at 1.48−1.49 g in the MMF plus 50% CNI reduction group and 1.54−1.55g in the MMF plus at least 75% CNI reduction group from week 2 through the end of the study.

**Figure 3:**
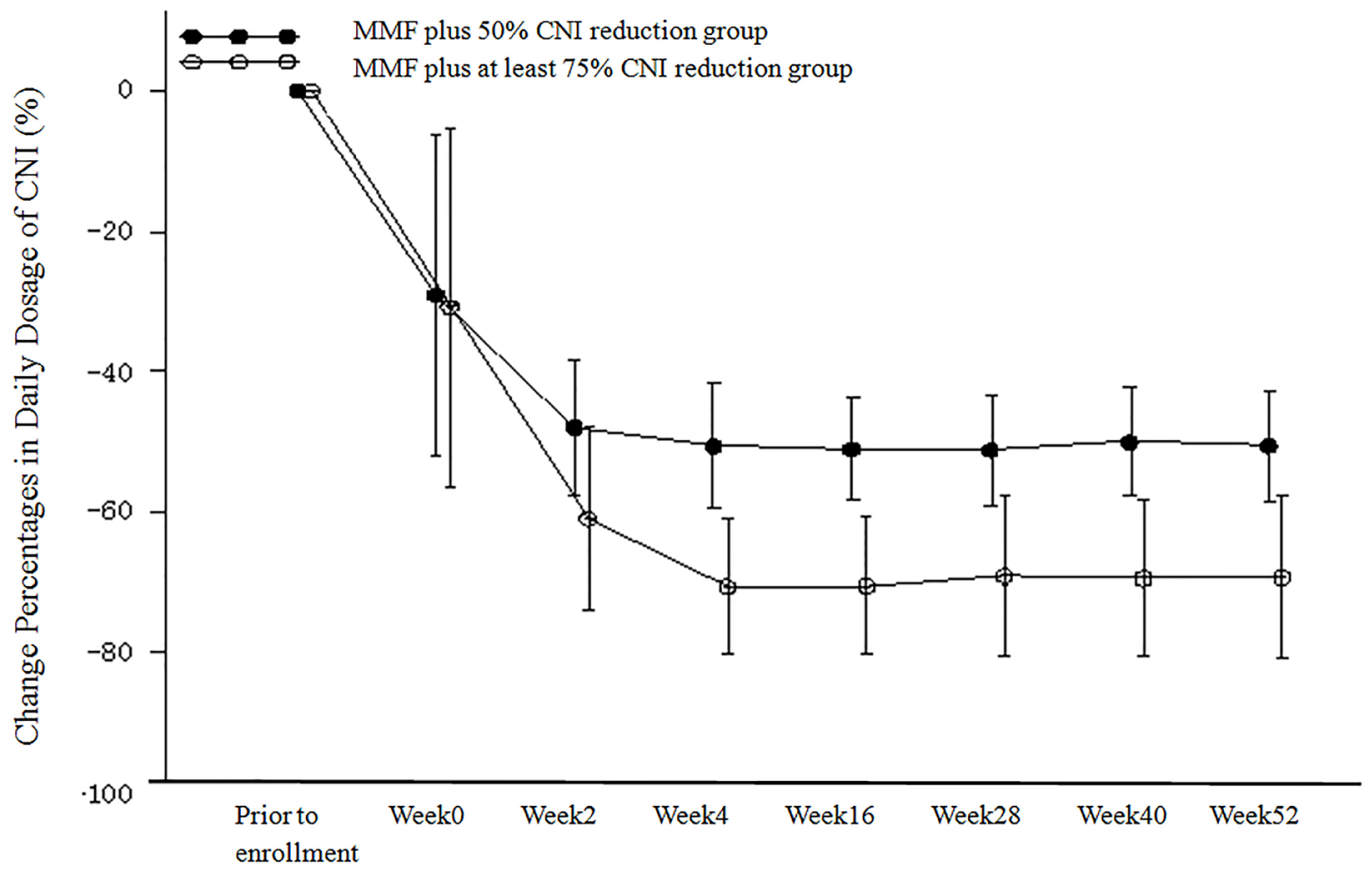
Change Percentages from Baseline in Daily Dosage of CNI (%) (PP Population)

A transition diagram showing the percent change in the daily dose of CNI (FK506/CyS) relative to baseline in the PP population is shown in Figure 3. The daily dose of CNI was reduced in both treatment groups according to the indicated protocol. The mean percentage reduction of the CNI daily dose from baseline in the MMF plus 50% CNI reduction group was 29.09% at week 0, 47.97% at week 2, 50.51% at week 4 (which achieved the target reduction percentage [i.e. 50%]), and was stable at approximately 50% thereafter. The mean percentage reduction from baseline of the daily dose of CNI in the MMF plus at least 75% CNI reduction group was 30.81% at week 0, 60.99% at week 2, and was stable at approximately 70% from week 4 until the end of the study. Thus, the actual mean percentage reduction from baseline of the CNI daily dose in the MMF plus at least 75% CNI reduction group was 70%.

### Efficacy

No patient in the MMF plus at least 75% CNI reduction group exhibited a decline in renal function 1 year after regimen adjustment. Two out of 37 patients (5.4%) in the PP population and 3 out of 43 patients (7%) in the ITT population in the MMF plus 50% CNI reduction group showed a reduction in renal function 1 year after regimen adjustment. There were no statistically significant differences between the two treatment groups (*P* = 0.494 in PP population, and *P* = 0.242 in ITT population, respectively). There was no graft loss or patient death in either treatment group during the study. No patient in the MMF plus 50% CNI reduction group experienced clinically suspected or biopsy-proven acute rejection during the 52 weeks after regimen adjustment. Two out of 34 patients (5.9%) in the PP population and 3 out of 40 patients (7.5%) in the ITT population in the MMF plus at least 75% CNI reduction group experienced at least one clinically suspected or biopsy-proven acute rejection during the 52 weeks after regimen adjustment. There were no statistically significant differences between the two treatment groups (*P* = 0.240 in the PP population).

**Figure 4:**
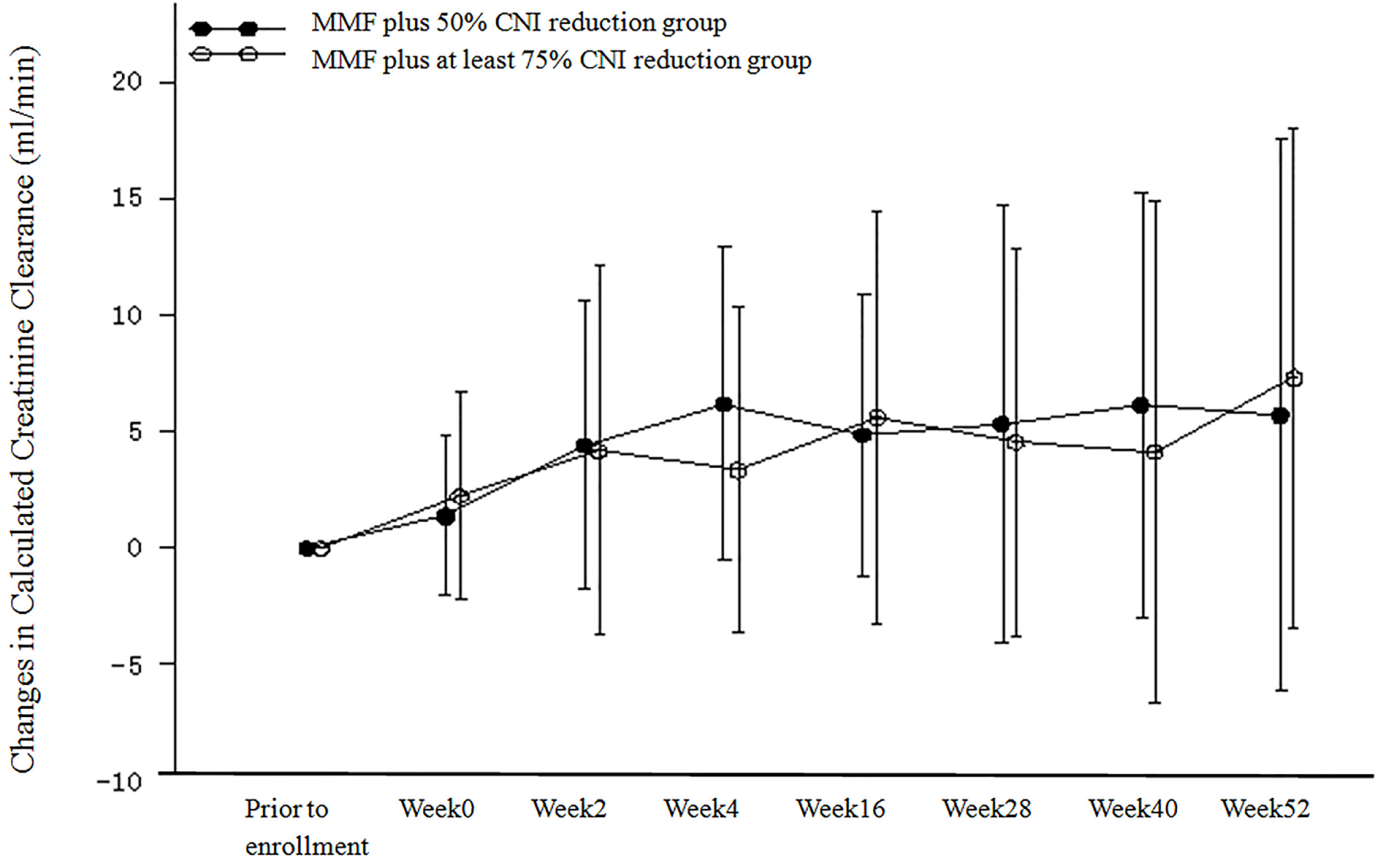
Changes from Baseline in Calculated Creatinine Clearance (ml/min) (PP Population)

We calculated the changes in creatinine clearance from baseline in the PP population (Table 3 and Figure 4). Creatinine clearance increased after regimen adjustment in both treatment groups. The changes were statistically significant at all time points with the exception of week 40 in the MMF plus at least 75% CNI reduction group. There were no statistically significant differences in creatinine clearance between the two treatment groups at all time points. Similar results were obtained for the ITT population. An analysis of covariance model was used to evaluate creatinine clearance at week 52, with the creatinine clearance at baseline as the covariate (due to the fact that the calculated creatinine clearance at baseline in the two treatment groups was not comparable). The results for the PP population indicated that the corrected mean change in creatinine clearance relative to baseline at week 52 was 6.551 mL/min in the MMF plus 50% CNI reduction group and 6.442 mL/min in the MMF plus at least 75% CNI reduction group. The difference in the creatinine clearance rate and the 95% confidence intervals were 0.110 mL/min and (−5.342−5.561) mL/min. Similar results were obtained for the ITT population.

**Table 3:** Changes from Baseline in Calculated Creatinine Clearance [ml/min] (PP Population)

Time point	MMF plus 50% CNI Reduction *N*= 37		MMF plus at least 75% CNI Reduction *N*= 34		*P* value between groups
	*N*	Mean(SD)	*N*	Mean(SD)	
Week 0	36	1.385(3.433)*	31	2.250(4.469)**	*P* = 0.374
Week 2	34	4.410(6.163)***	29	4.178(7.901)**	*P* = 0.896
Week 4	32	6.198(6.745)***	32	3.357(6.981)*	*P* = 0.103
Week 16	37	4.884(6.016)***	33	5.629(8.825)***	*P* = 0.685
Week 28	34	5.333(9.365)**	33	4.560(8.325)**	*P* = 0.722
Week 40	35	6.131(9.148)***	27	4.125(10.802)	*P* = 0.432
Week 52	37	5.715(11.838)**	34	7.328(10.727)***	*P* = 0.551

Comparison within group: *P < 0.05, **P < 0.01, ***P < 0.001

**Figure 5:**
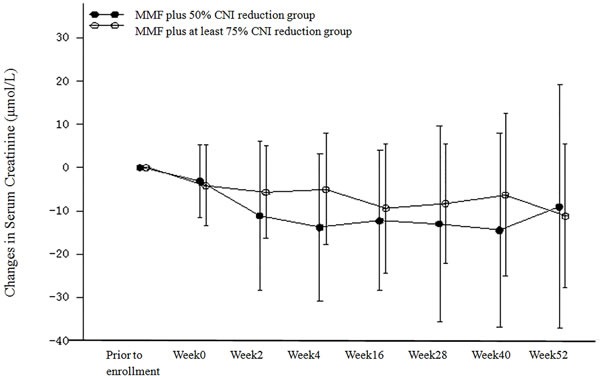
Changes from Baseline in Serum Creatinine (μmol/L) (PP Population)

The analysis of the changes in serum creatinine in the PP population are shown in Table 4 and Figure 5. Serum creatinine decreased in both treatment groups after regimen adjustment. There were no statistically significant differences in the mean serum creatinine between the two treatment groups at all time points with the exception of week 4. At week 4, the mean decrease in serum creatinine was higher in the MMF plus 50% CNI reduction group (13.863 μmol/L) than in the MMF plus at least 75% CNI reduction group (5.044 μmol/L) [*P* = 0.023]. The mean decrease from baseline in serum creatinine at week 52 was 9.078 μmol/L in the MMF plus 50% CNI reduction group and 11.262 μmol/L in the MMF plus at least 75% CNI reduction group. Similar results were obtained for both the ITT and PP populations.

**Table 4:** Changes from Baseline in Serum Creatinine [μmol/L] (PP Population)

Time point	MMF plus 50% CNI Reduction *N* = 37		MMF plus at least 75% CNI Reduction *N* = 34		*P* value between groups
	*N*	Mean(SD)	*N*	Mean(SD)	
Week 0	36	−3.175(8.536)*	31	−4.229(9.418)*	*P* = 0.633
Week 2	34	−11.150(17.406)***	29	−5.693(10.673)**	*P* = 0.133
Week 4	32	−13.863(16.981)***	32	−5.044(12.919)*	*P* = 0.023
Week 16	37	−12.316(16.216)***	33	−9.406(14.902)***	*P* = 0.439
Week 28	34	−13.041(22.599)**	33	−8.309(13.853)**	*P* = 0.304
Week 40	35	−14.480(22.396)***	27	−6.337(18.781)	*P* = 0.134
Week 52	37	−9.078(28.194)	34	−11.262(16.701)***	*P* = 0.690

Comparison within group: *P < 0.05, **P < 0.01, ***P < 0.001

### Safety

During the study, 16 patients (37.2%) in the MMF plus 50% CNI reduction group experienced a total of 36 AEs, and 17 patients (42.5%) in the MMF plus at least 75% CNI reduction group experienced a total of 35 AEs. There were no statistically significant differences between the two treatment groups (*P* = 0.659 for the ITT population). AEs were analyzed using the System Organization Class (SOC) hierarchy (Table 5). The most common AE was an investigation abnormality; There were seven patients (16.3%) who experienced a total of 17 AEs in the MMF plus 50% CNI reduction group, and nine patients (22.5%) who experienced a total of 18 AEs in the MMF plus at least 75% CNI reduction group. The less common AEs included infections [five patients (11.6%) who experienced a total of six AEs in the MMF plus 50% CNI reduction group, and five patients (12.5%) who experienced a total of six AEs in the MMF plus at least 75% CNI reduction group]. Gastrointestinal events were frequently reported in the MMF plus 50% CNI reduction group [3 patients (7.0%), 6 events total]. One patient (2.5%) in the MMF plus at least 75% CNI reduction group experienced a gastrointestinal event.

**Table 5:** Analysis of Adverse Events by SOC (ITT Population)

System Organization Class (SOC) Preferred Term (PT)	MMF plus 50% CNI Reduction (*N*= 43)	MMF plus at least 75% CNI Reduction (*N*= 40)
	*N* (%)	*N* (%)
Any adverse event	16(37.2)	17(42.5)
Investigations	7(16.3)	9(22.5)
Alanine aminotransferase increased	1(2.3)	4(10.0)
White blood cell count decreased	4(9.3)	1(2.5)
Aspartate aminotransferase increased	1(2.3)	3(7.5)
Blood creatinine increased	2(4.7)	2(5.0)
Blood urine present	2(4.7)	1(2.5)
Blood glucose increased	0(0.0)	1(2.5)
Gamma-glutamyltransferase increased	0(0.0)	1(2.5)
Glucose urine present	0(0.0)	1(2.5)
Neutrophil count decreased	0(0.0)	1(2.5)
Red blood cell count decreased	1(2.3)	0(0.0)
Weight decreased	1(2.3)	0(0.0)
Urine leukocyte esterase positive	1(2.3)	0(0.0)
Protein urine present	1(2.3)	0(0.0)
Infections and infestations	5(11.6)	5(12.5)
Upper respiratory tract infection	2(4.7)	2(5.0)
Urinary tract infection	2(4.7)	0(0.0)
Gastroenteritis	1(2.3)	0(0.0)
Hepatitis B	0(0.0)	1(2.5)
Lung infection	1(2.3)	1(2.5)
Biliary tract infection	0(0.0)	1(2.5)
Gastrointestinal disorders	3(7.0)	1(2.5)
Diarrhoea	2(4.7)	1(2.5)
Mouth ulceration	2(4.7)	0(0.0)
Abdominal pain	1(2.3)	0(0.0)
Nausea	1(2.3)	0(0.0)
Immune system disorders	0(0.0)	2(5.0)
Transplant rejection	0(0.0)	2(5.0)
Neoplasm	0(0.0)	0(0.0)
Hepatic cancer metastatic	0(0.0)	1(2.5)

A total of four patients (4.8%) experienced serious AEs (SAEs). One patient (2.3%) in the MMF plus 50% CNI reduction group developed a lung infection. Three patients (7.5%) in the MMF plus at least 75% CNI reduction group experienced SAEs: transplant rejection and biliary tract infection (1 patient), recurrence of hepatitis B virus (1 patient), and hepatic cancer bladder metastasis (1 patient). There were no statistically significant differences between the two treatment groups (*P* = 0.348 in the ITT population). No deaths were reported during the study period. Three (7.0%) patients in the MMF plus 50% CNI reduction group and two (4.5%) in the MMF plus at least 75% CNI reduction group experienced AEs that lead to discontinuation. Only one patient (2.5%) in the MMF plus 50% CNI reduction group developed a new malignant tumor (hepatic cancer bladder metastasis). No other patients developed new-onset hepatic disease, post-transplant diabetes mellitus, hypertension, or hyperlipidemia.

## DISCUSSION

Our results are consistent with those of previous studies, which have demonstrated the efficacy of MMF combined with CNI dose reduction for preventing renal dysfunction in LT recipients [12, 21, 22, 25, 27]. We observed improvements in both serum creatinine levels and creatinine clearance after converting patients who had renal dysfunction after liver transplantation to MMF combined with a 50% or at least 70% reduction in CNI dose (the actual mean reduction percentage in the CNI daily dose from baseline in the MMF plus at least 75% CNI reduction group was 70%). The mean decrease in serum creatinine at week 52 in the PP population was 9.078 μmol/L in the MMF plus 50% CNI reduction group, and was 11.262 μmol/L in the MMF plus at least 75% CNI reduction group. The corrected mean increase in creatinine clearance at week 52 in the PP population was 6.551 mL/min in the MMF plus 50% CNI reduction group, and 6.442 mL/min in the MMF plus at least 75% CNI reduction group. In the PP population, only two patients (5.4%) in the MMF plus 50% CNI reduction group experienced a decline in renal function (defined as a greater than 20% decrease in the GFR [28]). These results were indicative of an improvement in renal function.

Pageaux et al. [12] defined CNI-related renal dysfunction as a persistent increase in the SCr level (> 140 μmol/L and < 300 μmol/L) on at least two successive occasions at least 1 month apart, proteinuria (< 1 g/24 hours), absence of hematuria, absence of renal arteries stenosis, or urinary tract disease. We lowered the SCr value cutoff from 140 μmol/L to 110 μmol/L because an SCr above 110 μmol/L was considered renal dysfunction at some centers. Although we did not put include all of these parameters in the exclusion criteria, we confirmed that all the enrolled patients had CNI-related renal dysfunction according to our definition.

Caroline et al. prospectively studied 49 LT recipients who were treated with CNIs and who developed CNI-associated chronic renal failure. MMF was administered to these patients and the CNI dose reduced or withdrawn [22]. The increase in creatinine clearance after 1 year from 42.9 ± 14 mL/min at baseline to 48.8 ± 17 mL/min and extent of improvement were similar to the results of our study. However, the decrease or withdrawal of CNI resulted in episodes of acute graft rejection, which ranged from 9%-38% after CNI withdrawal [21, 24, 29]. Reich et al. [21] reported that two out of 16 patients (11%) had a 50% reduction in CNI dose and 6 out of 20 patients (30%) had a total withdrawal of CNI, which resulted in acute rejection of the liver graft. In this study, only two out of 34 patients (5.9%) in the PP population in the MMF plus at least 75% CNI reduction group experienced acute rejection during the year after regimen adjustment. The results showed that the incidence of acute rejection was relatively low, even when the CNI dose was reduced by 50%-70%, indicating MMF (1.5 g/d) was relatively safe and had a nephroprotective effect. Finally, none of the patients had graft failure or died during the study.

Creput et al. demonstrated that the improvement in renal function after MMF treatment was greater when CNI was totally, as opposed to partially, withdrawn [22]. However, different studies have reported conflicting results. In our study, no statistically significant differences in serum creatinine or creatinine clearance relative to baseline were observed between the two treatment groups. The two treatment groups were comparable in terms of the occurrence of a 20% or greater decrease in creatinine clearance relative to baseline, acute rejection, graft loss, or death during the year following regimen adjustment. These results indicated that the efficacy of MMF plus a 50% reduction in CNI dose was comparable to that of MMF plus at least a 70% reduction in CNI dose in LT patients with renal dysfunction. Our assessment of AEs, the onset of new diseases/infections, and clinical laboratory parameters revealed that MMF combined with a 50% or at least 70% reduction in CNI dose was safe and tolerable.

Declined renal function and acute rejection were observed separately in some cases. In the MMF plus 50% CNI reduction group, two out of 37 patients (5.4%) in the PP population experienced a decline in renal function, but no patients experienced acute rejection 1 year after regimen adjustment. In the MMF plus at least 75% CNI reduction group, two out of 37 patients (5.9%) in the PP population experienced acute rejection, but no patients experienced a decline in renal function 1 year after regimen adjustment. Given the limited sample size, although the differences in the occurrence of declined renal function or acute rejection between the two groups were not statistically significant, some trends were observed (i.e. compared to the MMF plus 50% CNI reduction group, the occurrence of declined renal function in the MMF plus at least 75% CNI reduction group was lower, but the occurrence of acute rejection in the MMF plus at least 75% CNI reduction group was higher). The results suggest that physicians should consider renal function and the risk of acute rejection when selecting the appropriate CNI reduction program.

In summary, for LT recipients with chronic renal dysfunction, conversion to MMF in combination with a 50% or at least 70% dose reduction in CNI could improve renal function. There were no significant differences between the 50% and at least 70% CNI reduction regimens in efficacy or safety.

## MATERIALS AND METHODS

### Study design

This was an open label, prospective, randomized, multi-centered controlled study of patients who developed chronic renal dysfunction after LT and treatment with a CNI-based immunosuppressive regimen. The study was performed at nine centers in China between June 2008 and July 2011. The study protocol was approved by the Institutional Review Board at each center. All patients provided written informed consent. The study was conducted in accordance with the principles of Good Clinical Practice and the Declaration of Helsinki. The study was registered at ClinicalTrials.gov (NCT00717314).

### Patients

Patients were required to meet all of the inclusion criteria at least 3 months after transplantation. The inclusion criteria were the following: 1) male or female liver allograft recipient of at least 18 years of age; 2) single organ recipient of a liver allograft; 3) treatment with a CNI-based immunosuppressive regimen prior to entry into the study; 4) serum creatinine measurement within the 30 days prior to regimen adjustment; 5) at least three months post-LT with renal dysfunction [serum creatinine of 110-300 μmol/L at entry (an SCr above 110 μmol/L could be diagnosed as renal dysfunction at some centers)]; and 6) negative pregnancy test for women of childbearing potential with reliable contraception (contraceptives must have been taken prior to beginning therapy with the study drugs, during therapy, and for 6 weeks after the last dose). The exclusion criteria were: 1) last recorded calculated creatinine clearance < 20 mL/min prior to regimen adjustment; 2) female patients who were pregnant or lactating; 3) diagnosis with any form of substance abuse, psychological illness, or any other condition that could interfere with the ability to understand the requirements of the study; 4) treatment with another investigational drug within the 30 days prior to enrollment, treatment with another immunosuppressant medication (approved or prohibited) before or after LT; 5) contraindications for CNI, corticosteroids, or MMF; 6) not available for routine study visits or follow-up at an accredited laboratory.

Before randomization, a reassessment of eligibility was performed and a detailed medical history was obtained. The open-label treatment period consisted of visits two to seven (weeks 2, 4, 16, 28, 40, and 52). Randomization was performed with a computer-generated randomized table at each center. During the treatment period, the following clinical tests were performed: blood chemistry, hepatic enzyme levels, trough levels of CNI, GFR (calculated with the 6-variable Modification of Diet in Renal Disease equation [23]), creatinine clearance (calculated using the Cockcroft and Gault method [30]), serum virology detection, and the CNI/MMF whole blood trough level. Patients were monitored for evidence of acute rejection or graft loss at every visit. Patients had the right to withdraw from the study at any time for any reason. The investigator also had the right to withdraw patients from the study if it was in the best interests of the patient, or for any of the following reasons: AEs, intercurrent disease, unsatisfactory therapeutic response (e.g. graft loss, excessive acute rejection, or re-transplantation), non-compliance with the protocol, or other reasons.

### Treatment regimens

Recipients who fulfilled the entry criteria were randomized into the study within 2 days of screening, and received one of the following immunosuppressive therapy regimens: MMF combined with a 50% reduction in CNI dose from baseline, and MMF combined with at least a 75% reduction in CNI dose from baseline. The MMF dose was 1.5-2.0 g/d, bid. Patients were treated according to this regimen for 52 weeks. Patients in the MMF plus 50% CNI reduction group received a reduced CNI dose. The dose of CNI was reduced by 25% within 1 week of entry into the study, and to 50% after 2 weeks. Patients in the MMF plus at least 75% CNI reduction group also received a reduced CNI dose. The CNI dose was reduced to 50% within two weeks of entry into the study and was reduced by more than 75% after 2 weeks.

### Study endpoints

The primary study endpoint was declined renal function, which was defined as greater than a 20% decrease in the calculated GFR during the year following regimen adjustment. The secondary endpoints are shown in Table 1.

### Statistical analysis

We aimed to identify immunosuppressive regimens that could reduce the long-term adverse effects of medications including nephrotoxicity while preventing acute rejection. A 10 mL/min difference in the GFR at month 12 between the two treatments was considered clinically relevant. As far as the primary endpoint was concerned (mean GFR at week 52), the common standard deviation within the group was assumed to be 19 mL/min. Differences between groups were analyzed using two-sided tests. A P value of 0.05 was the threshold for statistical significance. A sample of 72 patients achieved 90% power to detect a difference in the mean GFR of 10 mL/min. We planned to enroll 90 patients in order to obtain 36 patients that could be evaluated in each group.

The primary and secondary endpoints were analyzed in both an ITT population (all patients included in the study who received at least one dose of the treatment group-specific medication), and PP population (a subset of the ITT population, which included patients who did not drop out of the study prematurely). Analysis of variance was performed to analyze the effects of treatment on the primary endpoint. The secondary endpoints were analyzed in an explorative manner and using graphical methods and statistical tests as appropriate. The efficacy assessment was primarily based on the PP population. The significance level (α) of the statistical tests was 5%, and an alternative test was two-sided. Corresponding 1-α confidence intervals were calculated for the main efficacy parameters.
